# The comparison of predictive value of cervical length in singleton spontaneous preterm labor with in vitro fertilization pregnancies: A cohort study

**DOI:** 10.18502/ijrm.v19i1.8180

**Published:** 2021-01-25

**Authors:** Razieh Mohammad Jafari, Mahvash Zargar, Mojgan Barati, Somayeh Ershadian

**Affiliations:** Fertility Infertility and Perinatology Research Center, Ahvaz Jundishapur University of Medical Sciences, Ahvaz, Iran.

**Keywords:** Cervix, IVF, Preterm delivery, PTL.

## Abstract

**Background:**

Preterm labor (PTL) is one of the most important factors in neonatal mortality. Some studies have revealed a reverse relationship between cervical length (CL) and PTL, however, further evidence is needed to confirm it.

**Objective:**

To investigate the predictive value of CL in spontaneous and in vitro fertilization (IVF) pregnancies.

**Materials and Methods:**

This prospective cohort study was performed on 154 pregnant women from 16-26 wk of gestation with singleton fetus in spontaneous delivery (n = 77) and IVF pregnancies (n = 77) and followed up until delivery. Women with multiple pregnancy, placenta previa, cerclage, and congenital anomalies were excluded from study. The cut-off determination was done according to the Roc analysis.

**Results:**

The mean CL in term delivery and PTL groups were 37 ± 7 mm and 31 ± 6 mm, respectively (p < 0.001). The frequency of PTL in spontaneous and IVF pregnancies were 7.8% and 23.27%, respectively (p = 0.007). According to the Roc analysis, the best cut-off for normal pregnancy was ≤ 36 mm with the negative predictive value of 97.9%, the positive predictive value of 11.4%, sensitivity 83.3%, and specificity of 46.5%. While for the IVF group, the cut off was ≤ 30 mm, with a negative predictive value of 88.4%, positive predictive value of 57.8%, sensitivity of 63.2%, and specificity of 86%.

**Conclusion:**

In this study, IVF had a significant direct correlation with PTL. CL also had a significant indirect relationship with PTL.

## 1. Introduction

Preterm delivery (PTD) is a significant cause of neonatal morbidity and mortality. Over the last two decades, a worldwide increase has been seen in the rate of PTD (1). PTD is comprised of 10% of deliveries; it causes 75% of perinatal mortalities. However, owing to the improvement in the premature neonatal care, the morbidity and mortality resulting from preterm labor (PTL) has decreased. The increased rate of PTL may be due to the increase in the usage of infertility therapies and assisted reproduction technologies and increased age of pregnancy. Transvaginal ultrasound (TVU) for the measurement of cervical length (CL) has been evaluated in both asymptomatic and symptomatic women in terms of PTL (2). The correlation between a short CL and PTD is well established in singleton pregnancies and even the shortened cervix is a better predictor for PTD than clinical examination or fetal fibronectin (3, 4). Also, compared to normal singleton pregnancies, in vitro fertilization (IVF)-conceived singleton pregnancies is associated with higher pregnancy-related complications, including increased risk of cesarean delivery, intrauterine growth restriction, preeclampsia, congenital malformations, low birth weight, and higher risk of PTD (5). Similarly, PTD is more prevalent in IVF-conceived pregnancies as compared with those conceived naturally (6). A meta-analysis of 15 studies showed that the risk of spontaneous preterm birth (PTB) in singleton pregnancies resulting from IVF is significantly higher than that in spontaneously conceived singletons (7). In high-risk women for PTL, the CL is short. Therefore, the CL measurement is used to predict higher-risk pregnancies (8). Data pertaining to the measurement of CL and specifically studies assessing whether the increased risk of spontaneous PTB is predicted by a difference in CL are limited.

Therefore, the aim of this study was to evaluate the predictive value of transvaginal sonographic CL for the prediction of PTD before 37 wk between two groups of spontaneous and IVF pregnancies in asymptomatic women.

## 2. Materials and Methods

This prospective cohort study included 154 singleton pregnant women who attended the Prenatal Care Clinic and Infertility Clinic at Imam Khomeini Hospital affiliated to the Jundishapur University of Medical Sciences between March 2016 and June 2017 in Ahvaz, Iran. Participants were divided into two groups of spontaneously (n = 77) and IVF-conceived (n = 77) pregnancies. Patients were enrolled at 16-26 wk of gestation and followed up until the delivery. The exclusion criteria of the study included women with multiple pregnancies, placenta previa, cerclage, fetal anomalies, and elective PTL. In both groups, CL was measured from 16 through 26 weeks of gestation using TVU. Sonography was performed by two sonographers who are certified by FMF for CL measurement (V20 Madison). For the TVU, the women were first asked to empty their bladder after which they were placed in the lithotomy position. The sonography probe was inserted in anterior vaginal fornix without pressure. Sagittal cervix was shown and linear distance between internal and external OS was measured. All patients were followed until delivery and demographic data, gestational age at delivery, and CL (mm) at 16-26 weeks of gestation were recorded. Gestational age was determined from the date of the last menstrual period (LMP) and confirmed by the measurement of the CRL. PTL was defined as delivery < 37 weeks of gestation in both groups. Pregnancy outcome was obtained from the interview with mothers. For the determination of the cut-off, the Roc analysis was used and the sensitivity, negative and positive predictive values were calculated.

### Ethical considerations

This study was approved by Ethics Committee of Ahvaz Jundishapur University of Medical Sciences (IRAJUMS.REC.1396.889). All participants signed an informed consent form prior to the study.

### Statistical analysis

All statistical analyses were performed using the Statistical Package for Social Sciences, version 18.0, SPSS Inc., Chicago, IL, USA (SPSS). The cut-off was determined according to the Roc analysis. The significance of differences between two groups was assessed using independent *t* tests and Chi square test. P < 0.05 was considered as statistically significant.

## 3. Results

A total of 154 pregnant women aged range of 19-46 yr with a mean age of 29.9 ± 4.9 yr in the normal group and 32.18 ± 5.58 yr in the IVF group (p = 0.052) were included in the study. Table I shows the demographic data and outcomes of the pregnancies. The average BMI in the spontaneous and IVF groups is shown in the table mentioned (Table I). The average birth's weeks in spontaneous group was significantly higher than the IVF group (p < 0.001). Six women in spontaneous group and 18 women in IVF group delivered before 37 weeks.

The mean CL did not significantly differ between while the mean CL did not differ significantly between the PTL and term pregnancies in the normal group, it was significantly lower in the PTL than the term pregnancies in the IVF group. Additionally, the mean CL in the PTL subgroup of spontaneous pregnancy was significantly greater than the mean CL in the PTL subgroup of IVF pregnancy (according to Table II).

The classification of CL according to Table III showed that all patients with CL < 25 mm were preterm, while all patients with CL ≥ 45 mm were term. Therefore, as presented in table IV, the best cut-off points obtained from the ROC curve were ≤ 36 mm for the spontaneous group, with a high negative predictive value (NPV) of 97.9%, low positive predictive value (PPV) of 11.4%, high sensitivity of 83.3%, and low specificity of 46.5% and ≤ 30 mm for the IVF group with a high NPV of 88.4%, low PPV of 57.8%, sensitivity of 63.2%, and low specificity of 86.0%.

According to tables V and VI, the number of cesarean deliveries in the IVF group was 20, of which 12 were term and 8 preterm. The number of cesarean deliveries in the spontaneous group was 10, with 9 term and 1 preterm.

The mean + SD CL for the all the subjects was 36.4 + 7.6 mm (range: 21-70 mm). The CL did not differ significantly between the two groups of IVF and spontaneously-conceived pregnancies (p = 0.06).

Of the 154 patients of the study, 130 (83.7%) were term and 24 (16.4%) PTL. The mean CL in the PTL group was significantly lower than that in the term group (Table III).

**Table 1:** Socio-demographic, CL, and PTL in the two groups of normal pregnancy and IVF


	**Spontaneous pregnancy (n = 77)**	**IVF pregnancy (n = 77)**	**P-value**
**Age (year)**	29.9 ± 4.9	32.2 ± 5.6	0.052
**BMI (kg/m${}^{2}$)**	26.6 ± 4.5	27.7 ± 6.5	0.4
**Gestational age (week)**	38.2 ± 1.2	36.7 ± 3.1	<0.001
**Measurement's week**	16.48 ± 1.6	18.48 ± 1.13	<0.001
**Underlying disease**	29 (37.6%)	12 (15.6%)	0.002
**CL (mm)**	37.5 ± 7.6	35.2 ± 7.6	0.06
**PTL**	6 (7.7%)	18 (23.4%)	0.014
Mean ± SD or n (%)			

**Table 2:** The CL in the normal pregnancies and IVF groups (according to the Roc analysis)


**Gestational age (wk)**	**Spontaneous**			**IVF**		
	**PTL**	**Term**	**P-value**	**PTL**	**Term**	**P-value**
**CL**	35.3 ± 3.1	37.5 ± 0.5	0.5	30.2 ± 6.9	36.7 ± 7.2	0.001

**Table 3:** Classification of pregnancies according to the CL in the term and preterm pregnancies


	**Term (n = 130)**	**PTL (n = 24)**	**P value**
**CL (mm)**		37 ± 7	31 ± 6	< 0.001
**CL category**				
	**< 25**	0 (0%)	5 (20%)	< 0.0001
	**25-29**	5 (3.9%)	6 (24%)	
	**30-34**	47 (36.4%)	5 (20%)	
	**35-39**	43 (33.3%)	6 (24%)	
	**40-44**	16 (12.4%)	3 (12%)	
	**45-49**	10 (7.8%)	0 (0%)	
	**≥ 50**	8 (6.2%)	0 (0%)	

**Table 4:** Parameters of accuracy for PTB (<37 weeks of gestation) based on TVU CL measurement at second trimester (according to the Roc analysis)


**Parameter of accuracy**	**Spontaneous pregnancies (Cut-off point ≤ 36 mm)**	**IVF pregnancies (Cut-off point ≤ 30 mm)**
**Sensitivity (%)**	83.3	63.2
**Specificity (%)**	46.5	86.0
**Positive predictive value (%)**	11.4	57.8
**Negative predictive value (%)**	97.9	88.4

**Table 5:** Type of delivery in IVF and spontaneous groups


	**Term**		**Preterm**	
	**C/S**	**VD**	**C/S**	**VD**
**IVF**	12 (20.3%)	47 (79.7%)	8 (44.4%)	10 (55.6%)
**Spontaneous**	9 (12.5%)	62 (87.5%)	1 (16.6%)	5 (83.4%)

**Table 6:** Causes of cesarean delivery in IVF and spontaneous groups


	**Patient request**	**Misrepresentation**	**Unresponsive of induction**	**Fetal distress in labor**
**IVF**	14	5	0	1
**Spontaneous**	4	3	1	2

## 4. Discussion

The predictive value of cervical measurement were compared via transvaginal sonography at second trimester (16-26 weeks) for predicting the PTB between the two groups of asymptomatic singleton pregnant women conceived spontaneously or by IVF. The ROC curve was used to suggest best cut-off rather than the percentile of CL measurement. The studies comparing the predictive value of CL for PTB between spontaneous and IVF singleton pregnancies are rare. Many studies have investigated the relationship between CL and PTB adopting different methodologies to obtain the cut-off point, either percentile or ROC curve.

The current study showed that the best cut-off points obtained from the ROC curve were ≤ 36 mm for the spontaneous group, with a high NPV of 97.9%, low PPV of 11.4%, high sensitivity of 83.3%, and low specificity of 46.5%, and ≤ 30 mm for the IVF group with high NPV 88.4%, low PPV of 57.8%, sensitivity of 63.2%, and low specificity of 86.0%. The most accurate cut-off for a screening test should have a high sensitivity and an adequate PPV and NPV (9). Therefore, our study has confirmed that the TVU CL measurement at the second trimester is a better screening test for spontaneous PTB in naturally conceived pregnancies compared to the IVF ones, since it has higher NVP (97.9% vs 88.4%, respectively).

In our study, the rate of PTB in the IVF group was significantly higher than that in the spontaneous group. These results are strongly in agreement with previously published studies and a large meta-analysis that have reported that the PTB is more prevalent in IVF-conceived pregnancies compare to those conceived spontaneously (5-7, 10, 11). Although, none of the studies so far has presented the clarification for such findings. However, we decided to find out whether IVF and naturally conceived pregnancies differ in CL measured by TVU in the second trimester. The only available meta-analysis showed that the decrease in the length of cervix is associated with the increase in the risk of PTB (12, 13). This conclusion was confirmed in all studies and also in the current study. However, we found that the mean CL did not significantly differ between the spontaneous and IVF groups (p = 0.06). The subgroups analysis showed that the length of cervix in the term and preterm pregnancies in the IVF group showed a significant difference (p = 0.001). In our study, none of the patients in the spontaneous group had a short cervix (CL < 25 mm), however, the rate of short cervix in the IVF group was 11.7%. A cut-off value of ≤ 36 mm was obtained in our study for predicting the PTB in spontaneous group which is greater than the values reported by other published studies. This discrepancy may be attributed to no case of short cervix in our study. While the lower cut-off point of CL (≤ 30 mm) suggested in our study for predicting PTB in IVF group can be attributed to the rate of short cervix of IVF patients (11.7%). Iams et al. published the first large study of CL measurement at 26 weeks of gestation in singleton pregnancies in 1996 (14). They reported cut-off points of 30, 25, and 20 mm related to the 25th, 10${}^{\mathrm{th}}$, and 5th percentile in their studied population according to the ROC curves.

## 5. Conclusion

In this study, IVF had a significant direct correlation with PTL. In addition, CL had a significant indirect relationship with PTL. The results of this study indicated that CL measurement in the second trimester of pregnancy in women with singleton baby has a lower cut-off for predicting PTD.

##  Conflict of Interest

The authors declare that there is no conflict of interest.
